# Impact of central venous pressure during the first 24 h and its time-course on the lactate levels and clinical outcomes of patients who underwent coronary artery bypass grafting

**DOI:** 10.3389/fcvm.2023.1036285

**Published:** 2023-05-23

**Authors:** Yu Zhao, Hongmin Zhang, Xiaoting Wang, Dawei Liu

**Affiliations:** Department of Critical Care Medicine, Peking Union Medical College Hospital, Peking Union Medical College, Chinese Academy of Medical Sciences, Beijing, China

**Keywords:** central venous pressure, 28-day mortality, lactate, coronary artery bypass grafting, lactate clearance

## Abstract

**Purpose:**

Previous studies have revealed that elevated mean central venous pressure (CVP) was associated with poor prognosis in specific patient groups. But no study explored the impact of mean CVP on prognosis of patients who underwent coronary artery bypass grafting surgery (CABG). The purpose of this study was to investigate the impacts of elevated CVP and its time-course on clinical outcomes of patients who underwent CABG and potential mechanisms.

**Methods:**

A retrospective cohort study was performed based on the Medical Information Mart for Intensive Care IV (MIMIC-IV) database. We first identified the CVP during specific period with the most predictive value. Patients were categorized into the low-CVP and high-CVP group on the basis of the cut-off value. A propensity score matching was used to adjust covariates. The primary outcome was a 28-day mortality. The secondary outcomes were 1-year mortality and in-hospital mortality, the length of intensive care unit (ICU) stay and hospitalization, acute kidney injury incidence, use of vasopressors, duration of ventilation and oxygen index, and lactate levels and clearance. Patients in the high-CVP group were categorized into the “second day CVP ≤ 13.46 mmHg” group and the “second day CVP > 13.46 mmHg” group, respectively, and the clinical outcomes were the same as before.

**Results:**

A total of 6,255 patients who underwent CABG were picked from the MIMIC-IV database, of which 5,641 CABG patients were monitored by CVP measurement during the first 2 days after ICU admission and 206,016 CVP records were extracted from the database. The mean CVP during the first 24 h was the most correlative and statistically significant for the 28-day mortality. The risk of the 28-day mortality was increased in the high-CVP group [OR 3.45 (95% CI: 1.77–6.70; *p* < 0.001)]. Patients with elevated CVP levels had worse secondary outcomes. The maximum of lactate levels and lactate clearance were also poor in the high-CVP group. For patients in the high-CVP group during the first 24 h, whose mean CVP during the second day lowered to less than the cut-off value, had better clinical outcomes.

**Conclusions:**

An elevated mean CVP during the first 24 h was correlated with poor outcomes in patients who underwent CABG. The potential mechanisms may be influencing the lactate levels and lactate clearance through the impact on afterload of tissue perfusion. Patients whose mean CVP during the second day dropped to less than the cut-off value had favorable prognosis.

## Introduction

Coronary artery bypass grafting (CABG) remains the main invasive revascularization therapies of coronary artery disease (CAD) management (1). Over 50 years, the number of CABG procedures increase significantly in the United States and European countries (2). With almost 200,000 procedures per year in the United States and 1 procedure per 250 inhabitants in Europe, CABG may be reasonable to improve survival in patients with multiple vessel lesions of CAD, diabetes mellitus, or left cardiac insufficiency (1). Patients undergoing CABG are generally older patients, and a large portion had complex comorbidities; perioperative and postoperative complications are increasingly common (3). Therefore, the importance of risk assessment for clinical outcomes following CABG is undisputed.

Central venous pressure (CVP) is affected by many factors, such as fluid volume status, cardiac function, intra-thoracic pressure, and intra-abdominal pressure. Elevated CVP heralds the decreasing venous return, high afterload of organ perfusion, and deteriorating microcirculation (4–6). In patients with septic shock, elevated CVP is related to increased mortality and worsening organ function (7, 8). The level of mean CVP is correlated with acute kidney injury (AKI) in septic patients (9). In patients with cardiovascular diseases, elevated CVP exacerbated the deterioration of renal function and increased the risk of all-cause mortality (10). For critically ill patients in intensive care unit (ICU), elevated CVP prolonged the length of ICU stay and deteriorated clinical outcomes (11).

Some prior studies have shown that CVP plays crucial roles in expediting acute renal dysfunction in patients who underwent cardiac surgery (12–16). Most studies focused on a CVP in a specific time point or a mean CVP during several hours or days, the comparison between the first CVP immediately upon ICU arrival and the mean CVP of different time quantum has not been considered into studies. Meanwhile, the relevance between the time-course of CVP level and the clinical outcomes of CABG patients and its potential mechanisms are also unexplored.

## Methods

### Study design

A retrospective cohort study was conducted based on the Medical Information Mart for Intensive Care IV (MIMIC-IV, version 1.0) database, which contains comprehensive medical records of patients admitted into the ICUs of Beth Israel Deaconess Medical Center between 2008 and 2019. One author had passed the Collaborative Institutional Training Initiative examination, obtained the certification (record ID: 38403375), and got access to MIMIC-IV database.

### Selection of participants

CABG procedures were selected from MIMIC-IV through the International Classification of Diseases 9th Edition (ICD-9) code. The inclusion criteria were as follows: (1) all patients who underwent CABG procedures; (2) adults (≥18 years of age) at ICU admission; and (3) patients with complete CVP records and other medical records.

### Variable extraction

The Structure Query Language (SQL) with PostgreSQL (version 9.6) was utilized for extracting data including the following: (1) all CVP records during ICU stay; (2) baseline characteristics including age, sex, weight, ethnicity, the sequential organ failure assessment (SOFA), the Simplified Acute Physiology Score II (SAPS II), and the Charlson comorbidity index; (3) comorbidities including myocardial infarction (MI), congestive heart failure (CHF), diabetes, liver disease, chronic renal disease, hypertension, and atrial fibrillation (AFIB); (4) mean levels of vital signs including temperature (°C), heart rate, mean arterial pressure (MAP), and respiratory rate during the first 24 h in the ICU; (5) laboratory parameters such as white blood cell (WBC) count, hemoglobin, platelet counts, bicarbonate, blood urea nitrogen (BUN), creatinine, lactate, partial pressure of oxygen (PO_2_), partial pressure of carbon dioxide (PCO_2_), and fraction of inspiration O_2_ (FiO_2_) during the first 24 h in the ICU; and (6) the data of mechanical ventilation, renal replacement therapy (RRT), and using of vasopressors were also collected. The AKI events were picked out by the Kidney Disease Improving Global Outcomes (KDGIO) criteria, which was defined by a 0.3 mg/dl increase of serum creatinine within 48 h or 50% increase from baseline creatinine during 7 days.

### Outcomes

The primary outcome was the 28-day mortality in the present study. The secondary outcomes included in-hospital and 1-year mortality; the length of ICU stay and the length of hospitalization; the frequency of AKI after ICU admission; the incidence of RRT; the incidence of vasopressor use; the maximum of lactate levels and lactate clearance; and the duration of ventilation and the oxygenation index.

### Statistical analysis

All continuous variables are presented as mean (SD) or median [interquartile range (IQR)] no matter of normally distributed and skewed distributed data. All categorical variables are presented as total numbers and percentages (%). Continuous variables were analyzed through the Student's *t*-test or the exact Mann–Whitney *U*-test. Categorical variables were analyzed through the *χ*^2^ test or Fisher's exact test.

Correlations between the mean CVP of different time quantum and 28-day mortality were estimated by Spearman's rank correlation coefficient. The mean CVP of 6, 12, 24, and 48 h was defined as 6 h-CVP, 12 h-CVP, 24 h-CVP, and 48 h-CVP, respectively. All other covariates, which included demographic characteristics, ethnicity, comorbidities, SOFA score, SAPS II score, Charlson comorbidity index, vital signs, and laboratory tests, were analyzed by the multivariable regression model. Receiver operating characteristic (ROC) analysis of mean CVP levels in the first 24 h was performed and resulted in a cut-off value through which patients were divided into the high- and low-CVP groups. Propensity score matching (PSM) was used to adjust the covariates between the high-CVP and low-CVP group. Therefore, a logistic regression model was used to estimate the propensity scores of the patient, and a caliper width of 0.01 was applied for 1:1 matching for these two groups. Standardized mean differences (SMDs) were calculated to assess the equilibrium of characteristics between the two groups. Variables can be considered as balance when SMDs are less than 0.1 (17). The cumulative mortality was estimated by the Kaplan–Meier analysis.

Furthermore, an additional analysis was conducted to investigate whether the time-course of CVP level influenced the clinical outcomes of CABG patients. Patients who have been in the high-CVP group in the first 24 h were categorized into the “second day CVP ≤13.46 mmHg” group and the “second day CVP >13.46 mmHg” group, respectively. These additional analysis methods were the same as before.

The IBM® SPSS® Statistics version 26 (SPSS Inc., Chicago, IL, United States) was used to perform all the statistical analyses, and a *p*-value <0.05 was regarded as statistically different.

## Results

### Baseline characteristics

**Figure 1:**
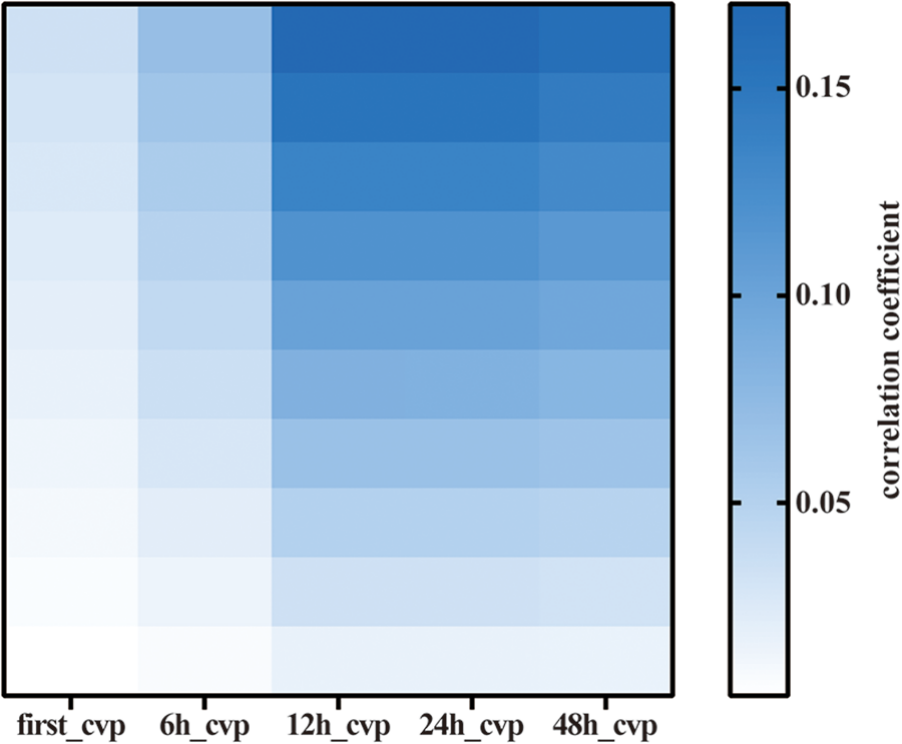
Heatmap of Spearman correlation analysis of various mean CVP. CVP, central venous pressure.

**Figure 2:**
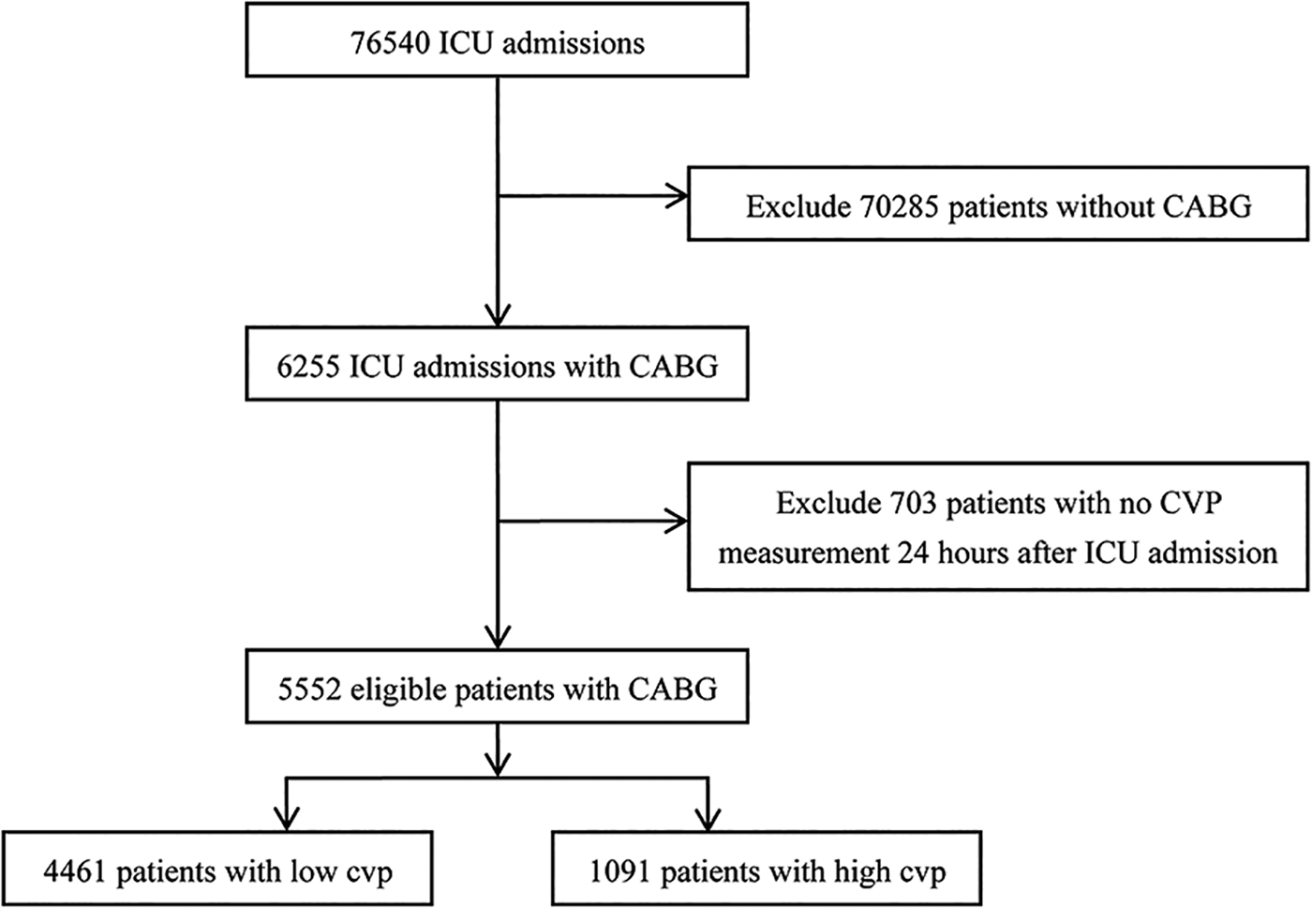
Study flow chart in the present study.

Among the 76,540 patients in the MIMIC-IV database, 6,255 adult patients underwent CABG, and 5,641 CABG patients had complete CVP records during the first 2 days; 206,016 CVP records were extracted from the database. We first compared the associations between first-CVP, 6h-CVP, 12h-CVP, 24h-CVP, 48h-CVP, and 28-day mortality (Figure 1). Spearman correlation coefficients suggested that the 12h-CVP and 24h-CVP are most correlative to 28-day mortality (Table 1). The logistic regression analyses demonstrated the predictive effects of the 24h-CVP and 48h-CVP on 28-day mortality (Table 1). We finally chose the 24h-CVP as the target of this study. Therefore, we exclude 703 patients with no CVP measurement 24 h after ICU admission. The flow chart showing the process of patient selections is presented in Figure 2. A total of 5,552 patients were included in this study (Supplementary Table S1), whose baseline characteristics and outcomes are shown in Supplementary Table S1.

**Table 1:** Spearman correlation coefficients and logistic regression *p*-values for various mean CVP of different time quantum-evaluated in the present study.

Various mean CVP	Correlation coefficient (r) with 28-day mortality	Strength of relationship	*p*-value	Logistic regression *p*-value
First-CVP	0.034	None	0.027	0.201
6h-CVP	0.070	Weak	<0.001***	0.025
12h-CVP	0.170	Weak	<0.001***	0.069
24h-CVP	0.170	Weak	<0.001***	0.001***
48h-CVP	0.161	Weak	<0.001***	0.001***

CVP, central venous pressure.

***p<0.001.

### Cut-off value and grouping

**Figure 3:**
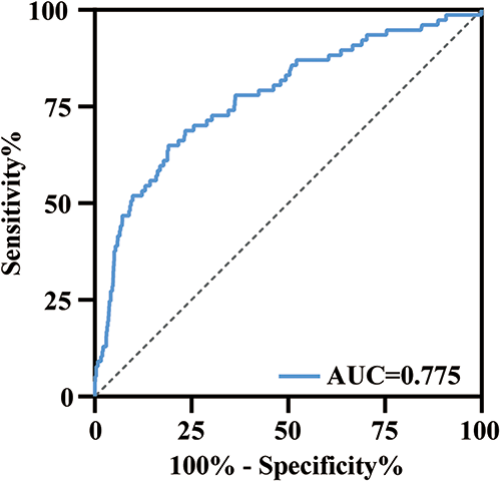
ROC curve analysis of 24h-CVP for 28-day mortality. The area under the curve was 0.775 (95% CI: 0.717–0.833, *p* < 0.001). ROC, receiver operating characteristic; AUC, area under the curve.

ROC curve was performed to find the cut-off value of the 24h-CVP in prediction of 28-day mortality (Figure 3). The area under the curve (AUC) for the 24h-CVP was 0.775 (95% CI: 0.717–0.833, *p* < 0.001), and the optimal cut-off value was 13.46 mmHg (Table 2). The 24h-CVP ≤ 13.46 was defined as the low-CVP group; conversely, the 24h-CVP >13.46 was defined as the high-CVP group. The baseline characteristics of the low-CVP group and high-CVP group are summed up in Table 3. Higher SOFA scores [7 (5–9) vs. 5 (4–7)] and SAPS II score [40 (32–49) vs. 35 (29–42)] were found in the high-CVP group.

**Table 2:** ROC analysis of 24h-CVP in the prediction of 28-day mortality.

	AUC	95% CI	*p*	Optimal cut-off	Sensitivity	Specificity	PPV	NPV
24h-CVP	0.775	0.717–0.833	<0.001	13.46	64.94%	80.96%	4.5%	99.39%

AUC, area under the curve; PPV, positive predictive value; NPV, negative predictive value; CVP, central venous pressure; ROC, receiver operating characteristic.

**Table 3:** Comparisons of baseline characteristics between the original cohort and matched cohort.

Covariates	Original cohort				Matched cohort			
	Low-CVP	High-CVP	SMD	*p*-value	Low-CVP	High-CVP	SMD	*p*-value
*N*	4,461	1,091			1,046	1,046		
Age	69 (62–76)	69 (61–77)	0.003	0.670	69 (62–76)	69 (62–77)	0.029	0.506
Male (%)	3,489/4,461 (78.2)	796/1,091 (73.0)	0.125	<0.001***	772/1,046 (73.8)	772/1,046 (73.8)	<0.001	0.999
Weight (kg)	84.0 (72.7–96.0)	88.0 (77.0–102.0)	0.260	<0.001***	87.5 (75.7–101.9)	88.0 (77.0–102.0)	0.013	0.596
Ethnicity, *n* (%)								
Asian	115/4,461 (2.6)	16/1,091 (1.5)	0.073	0.034*	22/1,046 (2.1)	16/1,046 (1.5)	0.042	0.413
Black	156/4,461 (3.5)	45/1,091 (4.1)	0.033	0.320	45/1,046 (4.3)	43/1,046 (4.1)	0.009	0.913
White	3,201/4,461 (71.7)	804/1,091 (73.7)	0.043	0.214	744/1,046 (71.2)	774/1,046 (74.0)	0.064	0.155
Latino	134/4,461 (3.0)	31/1,091 (2.8)	0.009	0.842	32/1,046 (3.0)	31/1,046 (3.0)	0.005	0.999
Other	855/4,461 (19.2)	195/1,091 (17.9)	0.033	0.343	203/1,046 (19.4)	182/1,046 (17.4)	0.051	0.259
Comorbidities, *n* (%)								
Myocardial infarction	1,719/4,461 (38.5)	477/1,091 (43.7)	0.106	0.002**	446/1,046 (42.6)	449/1,046 (42.9)	0.006	0.930
Congestive heart failure	991/4,461 (22.2)	414/1,091 (37.9)	0.362	<0.001***	381/1,046 (36.4)	375/1,046 (35.9)	0.012	0.820
Diabetes	1,868/4,461 (41.9)	476/1,091 (43.6)	0.035	0.305	474/1,046 (45.3)	459/1,046 (43.9)	0.029	0.538
Liver disease	136/4,461 (3.0)	73/1,091 (6.7)	0.191	<0.001***	54/1,046 (5.2)	62/1,046 (5.9)	0.033	0.504
Chronic renal disease	772/4,461 (17.3)	257/1,091 (23.6)	0.161	<0.001***	235/1,046 (22.5)	244/1,046 (23.3)	0.020	0.677
Hypertension	2,753/4,461 (61.7)	617/1,091 (56.6)	0.106	0.002**	592/1,046 (56.6)	592/1,046 (56.6)	<0.001	0.999
Atrial fibrillation	1,740/4,461 (39.0)	522/1,091 (47.8)	0.180	<0.001***	472/1,046 (45.1)	486/1,046 (46.5)	0.027	0.568
Severity of illness								
SOFA score	5 (4–7)	7 (5–9)	0.626	<0.001***	7 (5–9)	6 (5–9)	0.004	0.930
SAPS II score	35 (29–42)	40 (32–49)	0.379	<0.001***	38 (31–49)	39 (32–48)	0.037	0.228
Charlson comorbidity index	5 (4–6)	6 (4–7)	0.250	<0.001***	5 (4–7)	6 (4–7)	0.042	0.053
Vital signs								
MAP (mmHg)	58 (54–62)	57 (52–61)	0.196	<0.001***	58 (53–62)	57 (52–62)	0.117	0.056
Heart rate (bpm)	67 (61–74)	69 (62–76)	0.172	<0.001***	68 (62–74)	69 (62–76)	0.108	0.051
Temperature (°C)	36.3 (35.7–36.4)	36.1 (35.6–36.5)	0.026	0.204	36.2 (35.6–36.4)	36.1 (35.6–36.5)	0.023	0.360
Respiratory rate (bpm)	11 (9–13)	11 (9–13)	0.057	0.107	11 (9–13)	11 (9–13)	0.027	0.434
Laboratory tests								
WBC (×10^9^/L)	9.0 (7.0–12.5)	9.4 (7.2–13.0)	0.090	<0.001***	9.2 (7.1–13.1)	9.4 (7.2–12.9)	0.012	0.705
Hemoglobin (×10^12^/L)	11.5 (9.6–13.4)	11.4 (9.6–13.2)	0.029	0.350	11.3 (9.5–13.2)	11.4 (9.6–13.2)	0.027	0.546
Platelet (×10^9^/L)	180 (141–226)	189 (147–238)	0.118	<0.001***	178 (141–223)	189 (148–237)	0.160	0.051
Bicarbonate (mmol/L)	24 (23–26)	25 (22–27)	0.047	0.215	24 (22–26)	25 (22–27)	0.101	0.057
BUN (mg/dl)	17 (14–22)	18 (14–25)	0.223	<0.001***	18 (14–25)	18 (14–25)	0.052	0.923
Creatinine (mg/dl)	0.90 (0.80–1.10)	1.00 (0.80–1.30)	0.117	<0.001***	1.00 (0.80–1.27)	1.00 (0.80–1.27)	0.120	0.362

CVP, central venous pressure; SMD, standardized mean difference; SOFA, sequential organ failure assessment; SAPS II, simplified acute physiology score II; MAP, mean arterial pressure; WBC, white blood cell; BUN, blood urea nitrogen.

*p<0.05; **p<0.01; ***p<0.001.

### Primary outcome and secondary outcomes with propensity score-matched cohorts

**Figure 4:**
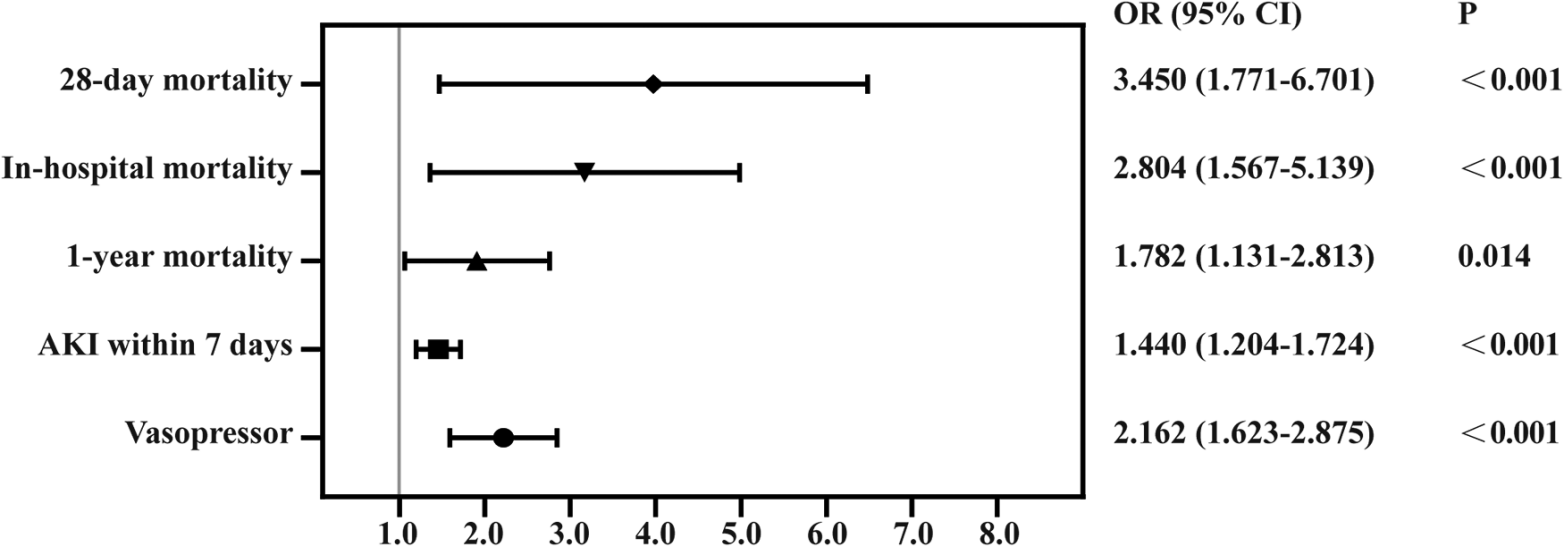
Forest plot for odds ratio of clinical outcomes in propensity score-matched cohorts. AKI, acute kidney injury.

**Figure 5:**
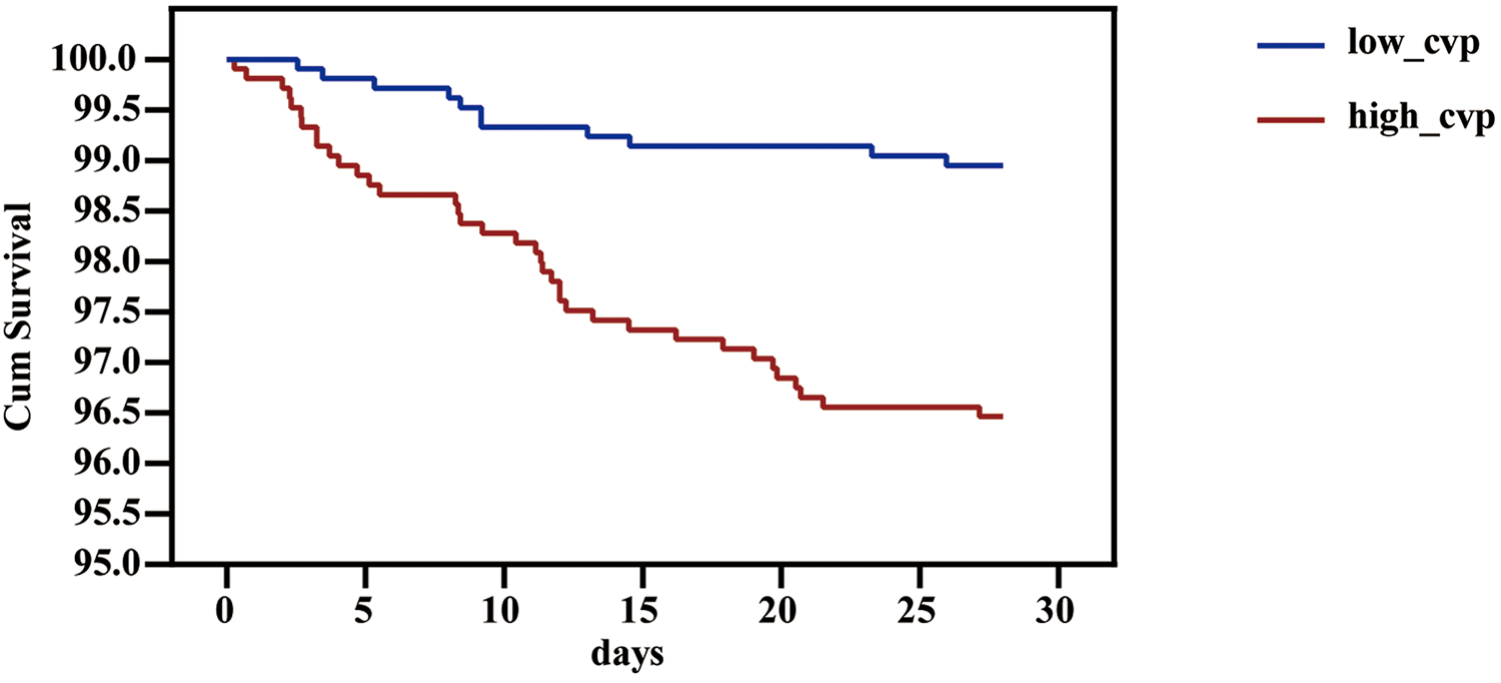
Survival curve of 28-day mortality of different 24h-CVP levels in the patients in the critical care unit. CVP, central venous pressure.

We used PSM to minimize the covariates imbalance between the low-CVP group and high-CVP group (Supplementary Figure S1). Matching was performed 1:1, and we finally acquired a matched cohort of 2,092 patients (Table 3). The high-CVP group had a higher 28-day mortality after PSM (Table 4 and Figure 4) (1.05% vs. 3.54%, *p* < 0.001) (OR: 3.450; 95% CI: 1.771, 6.701). The Kaplan–Meier's survival curve of 28-day mortality between different mean CVP levels is displayed in Figure 5. Higher mean CVP level during the first 24 h was also associated with higher in-hospital and 1-year mortality, and prolongation of length of ICU stay and hospitalization (Table 4 and Figure 4). Compared with the low-CVP group, the percentage of AKI within 7 days was significantly higher in the high-CVP group, but there were no significant differences on RRT incidences (Table 4). Lower mean CVP level during the first 24 h lowered the percentage of the use of vasopressor and inotropes (Table 4). Lower mean CVP level reduced the maximum of lactate levels on day 1 and improved lactate clearance (Table 4). Also, the low-CVP group was significantly associated with ameliorative oxygen index and shorter duration of ventilation (Table 4). By comparison with the high-CVP group, the fluid balance in the low-CVP group was significantly lower on day 1 and day 2 (Table 4).

**Table 4:** Clinical outcome analysis with propensity score-matched cohorts.

Outcomes	Low-CVP	High-CVP	*p*-value
Primary outcome			
28-day mortality	11/1,046 (1.05)	37/1,046 (3.54)	<0.001***
Secondary outcomes			
In-hospital mortality	15/1,046 (1.43)	41/1,046 (3.92)	<0.001***
1-year mortality	31/1,046 (2.96)	54/1,046 (5.16)	0.014*
Length of ICU stay (days)	2.09 (1.26–3.46)	2.39 (1.34–4.47)	<0.001***
Length of hospitalization (days)	7.85 (5.67–11.79)	8.71 (5.93–12.92)	0.006**
AKI within 7 days, *n* (%)	337/1,046 (32.22)	425/1,046 (40.63)	<0.001***
RRT, *n* (%)	29/1,046 (2.77)	45/1,046 (4.30)	0.075
Vasopressor, *n* (%)	889/1,046 (84.99)	967/1,046 (92.45)	<0.001***
Inotropes, *n* (%)	321/1,046 (30.69)	386/1,046 (36.90)	0.031**
Maximum of lactate level on day 1 (mmol/L)	3.02 (0.05)	3.42 (0.06)	<0.001***
Lactate clearance on day 1 (%)	15.99 (1.88)	9.20 (2.43)	0.033*
Duration of ventilation (h)	6.00 (3.82–16.00)	12.50 (5.00–23.13)	<0.001***
PO_2_/FiO_2_ (mmHg)	182 (133–242)	170 (117–232)	<0.001***
Fluid balance on day 1 (L)	9.10 (0.12)	10.03 (0.16)	<0.001***
Fluid balance on day 2 (L)	0.71 (0.08)	1.10 (0.10)	0.044*

CVP, central venous pressure; ICU, intensive care unit; AKI, acute kidney injury; RRT, renal replacement therapy; PO_2_, partial pressure of oxygen; FiO_2_, fraction of inspiration oxygen.

*p<0.05; **p<0.01; ***p<0.001.

### Additional analysis

**Figure 6:**
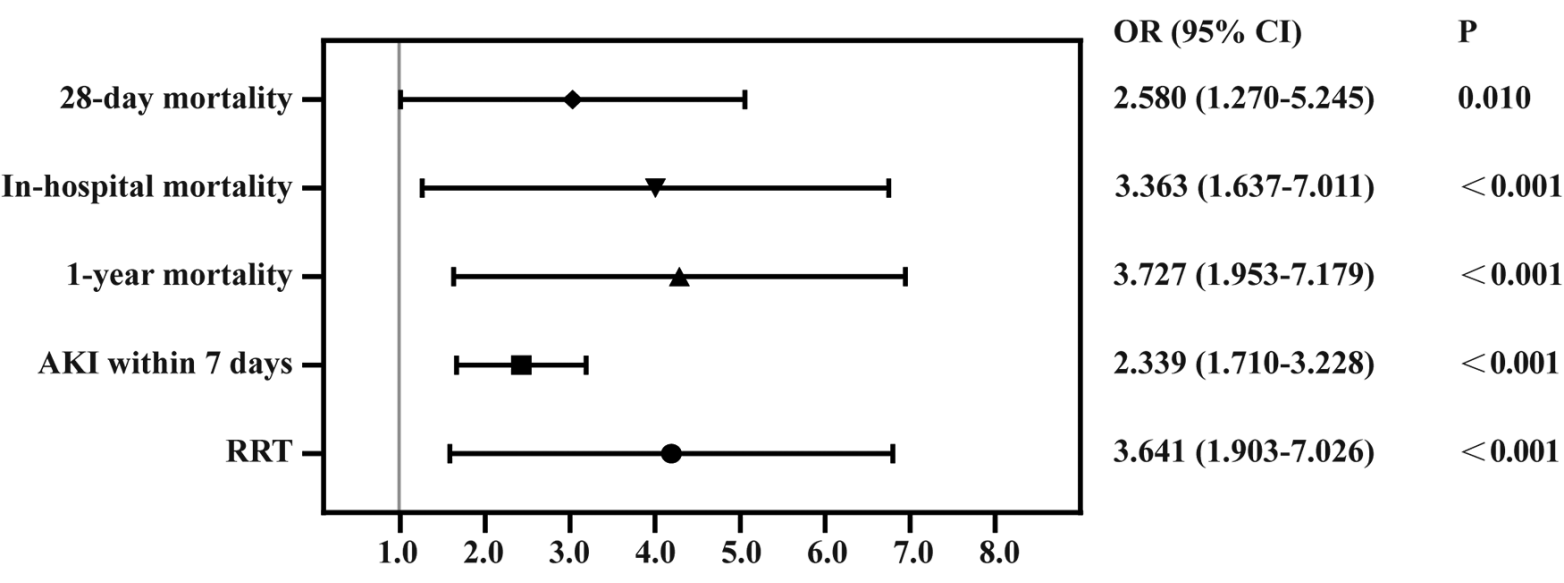
Forest plot for odds ratio of clinical outcomes in CVP dropped to less than 13.46 mmHg and group whose CVP did not drop to less than 13.46 mmHg. RRT, renal replacement therapy; AKI, acute kidney injury; CVP, central venous pressure.

A total of 1,091 patients had been categorized into the high-CVP group based on the mean CVP level during the first 24 h. According to the mean CVP level during the second day, we divided these patients into the “second day CVP ≤ 13.46 mmHg” group and the “second day CVP > 13.46 mmHg” group. The time-course of mean CVP level affects the prognosis of CABG patients. The “second day CVP > 13.46 mmHg” group showed higher risk of 28-day mortality, in-hospital mortality, 1-year mortality, AKI within 7 days, RRT events and worsening oxygen index and lactate levels, and prolongation of length of ICU stay, hospitalization, and ventilation (Table 5 and Figure 6).

**Table 5:** Clinical outcomes analysis in CVP dropped to less than 13.46 mmHg and group whose CVP did not drop to less than 13.46 mmHg.

Outcomes	Second day CVP ≤ 13.46 mmHg	Second day CVP > 13.46 mmHg	*p*-value
Primary outcome			
28-day mortality	10/275 (3.64)	33/372 (8.87)	0.010**
Secondary outcomes			
In-hospital mortality	9/ 275 (3.27)	38/372 (10.22)	<0.001***
1-year mortality	11/ 275 (4.00)	50/372 (13.44)	<0.001***
Length of ICU stay (days)	3.28 (2.14–5.22)	4.33 (2.63–8.87)	<0.001***
Length of hospitalization (days)	9.43 (6.39–14.47)	11.77 (7.67–17.73)	<0.001***
AKI within 7 days, *n* (%)	111/ 275 (40.36)	228/372 (61.29)	<0.001***
RRT, *n* (%)	11/ 275 (4.00)	49/372 (13.17)	<0.001***
Vasopressor, *n* (%)	263/ 275 (95.64)	363/372 (97.58)	0.1837
Maximum of lactate level on day 2 (mmol/L)	1.80 (0.08)	2.63 (0.16)	<0.001***
Lactate clearance on day 2 (%)	4.59 (2.77)	4.62 (2.85)	0.994
Duration of ventilation (h)	16.83 (7.60–29.98)	29.38 (14.25–104.62)	<0.001***
PO_2_/FiO_2_ (mmHg)	160 (106–221)	148 (104–107)	0.047*
Fluid balance on day 1 (L)	10.32 (0.33)	12.49 (0.31)	<0.001***
Fluid balance on day 2 (L)	1.08 (0.18)	3.08 (0.23)	<0.001***

CVP, central venous pressure; ICU, intensive care unit; AKI, acute kidney injury; RRT, renal replacement therapy; PO_2_, partial pressure of oxygen; FiO_2_, fraction of inspiration oxygen.

*p<0.05; **p<0.01; ***p<0.001.

## Discussion

Our study demonstrated that the mean CVP during the first 24 h after ICU admission is adequate to assess and predict the 28-day mortality rather than the mean CVP during shorter or longer time. The first CVP after ICU admission or mean CVP during 6 h or 12 h had no statistical significance on predicting 28-day mortality. The mean CVP during 48 h after ICU admission has nearly the same predictive effects as 24h-CVP. Lower 24h-CVP is also associated with lower in-hospital and 1-year mortality and lower risk of AKI within 7 days. Further exploration on the length of ICU stay and hospitalization, percentage of vasopressor use and duration of mechanical ventilation, and maximum of lactate level or lactate clearance related to tissue perfusion also showed that higher mean CVP level was related to poor ICU outcome. The time-course of CVP also affected the clinical outcomes of CABG patients, implying interventions that lower the CVP level could improve prognosis.

CVP is still the most widely used index to guide fluid resuscitation (18). According to the Frank–Starling curve, cardiac output ascends accompanied by CVP increase until a plateau is reached. But the application value is limited because prediction of fluid resuscitation from single CVP is meaningless (19). However, an “extreme” CVP value such as CVP less than 6 mmHg or greater than 15 mmHg could predict fluid responsiveness to a certain extent (20). In recent years, the trend of CVP and other hemodynamic parameters was considered comprehensively to titrate fluid therapy. The theory of maintaining CVP as low as possible in case of hemodynamic stabilization was realized by more and more specialists. On the contrary, elevated CVP is associated with increased mortality in patients with cardiovascular disease or sepsis (6, 10).

CVP is the pressure where superior and inferior vena cava enter into the right atrium, and it is composed of three factors: heart function, circulatory blood volume, and vessel tone. Volume overload state increased the venous return and resulted to elevated CVP. CVP is also influenced by the interaction of cardiac function and return function (21). Deteriorating cardiac dysfunction can also be the cause of elevated CVP. Right ventricular dysfunction increases the preload of the right ventricular and leads to the obstruction of venous return and elevated CVP. Our study demonstrated that more patients in the high-CVP group need vasopressors especially inotropes than patients in the low-CVP group, suggesting that CVP levels are associated with right ventricular dysfunction and hemodynamic stability. However, CVP is also influenced by intrathoracic pressure and intra-abdominal pressure, which makes it more complicated to interpret. In patients with pneumothorax, pericardial tamponade, and constrictive pericarditis, elevated CVP could be detected.

Based on Guyton's venous return theory, in case of constant mean circulatory filling pressure, elevated right atrial pressure decreased venous return and developed to systemic venous congestion (22), which increased “afterload” of tissue perfusion. In patients with chronic heart failure, high central venous pressure is an independent factor of predicting the worsening of renal function (23). A high CVP could also predict abnormal intrarenal flow dynamics, which is closely related to heart failure severity (24). In septic patients, there is a positive correlation of CVP levels and the risk of AKI, suggesting that venous congestion is a critical part in the development of AKI (9). In our study, patients with high CVP levels after CABG surgery had higher risk of AKI in 7 days.

Previous studies also demonstrated that higher CVP can affect the microcirculation. An elevated CVP leads to the impairment of microcirculatory perfusion, which demonstrates that the afterload of microcirculatory determined actual perfusion situation to some extent (6). In our study, we found that the level of CVP was related to the maximum of lactate levels, meanwhile a lower CVP associated with lower lactate levels. In addition, we also found that the CVP levels also affect the lactate clearance, confirming that a high CVP could attenuate microvascular perfusion.

Our previous studies showed the association of CVP and prognosis in patients with mechanical ventilation; elevated CVP indicated worse outcomes during the first 24 h of mechanical ventilation (25). Another study demonstrated that a high CVP was associated with prolonged mechanical ventilation and lower oxygen index (26). The high CVP levels might be associated with high PEEP levels and worse compliance of the respiratory system to some extent (26). The high CVP levels caused by volume overload could increase the risk of pulmonary edema and lower the PO_2_/FIO_2_ index. This study also confirmed high CVP indicating prolonged duration of ventilation and lower PO_2_/FIO_2_ index.

High CVP levels in the first 24 h indicated a high 28-day mortality. However, whether the second day mean CVP lowered to the cut-off value also affected clinical outcomes. The second day low-CVP group also had better prognosis. Our previous study also confirmed patients whose CVP dropped down within 1 week had a better 28-day survival rate (8), indicating that interventions which could lower CVP in time could improve prognosis.

There are several limitations in the present study. First, this study was a retrospective cohort study based on one center database. Therefore, we can just carry out correlation analysis rather than taking interventions to get causal relationships. Second, we only extracted data during the first 2 days after ICU admission, and extraordinary low or high CVP values may happen under certain pathophysiology processes. Third, multiple confounders that could affect clinical outcomes were unknown, such as clinical decisions and interventions before or after CVP measurement. Fourth, the surgical factors interfering CVP levels such as off-pump or on-pump methods and the operation duration were unknown. Another study investigating the potential factors of high CVP levels is needed in the future.

## Conclusion

In conclusion, an elevated mean CVP during the first 24 h was associated with poor clinical outcomes in patients who underwent CABG surgery. The first CVP or mean CVP during shorter period after ICU admission are meaningless for predicting prognosis. Patients whose mean CVP during the second day dropped to less than the cut-off value had a higher 28-day survival rate and favorable prognosis. The potential mechanisms may be influencing the afterload of tissue perfusion, which was reflected by lactate levels and lactate clearance.

## Data Availability

Publicly available data sets were analyzed in this study. These data can be found here: https://physionet.org/content/mimiciv/1.0/.
