# Corrigendum: Impact of central venous pressure during the first 24 h and its time-course on the lactate levels and clinical outcomes of patients who underwent coronary artery bypass grafting

**DOI:** 10.3389/fcvm.2023.1298638

**Published:** 2023-10-09

**Authors:** Yu Zhao, Hongmin Zhang, Xiaoting Wang, Dawei Liu

**Affiliations:** Department of Critical Care Medicine, Peking Union Medical College Hospital, Peking Union Medical College, Chinese Academy of Medical Sciences, Beijing, China

**Keywords:** central venous pressure, 28-day mortality, lactate, coronary artery bypass grafting, lactate clearance

A Corrigendum on Impact of central venous pressure during the first 24 h and its time-course on the lactate levels and clinical outcomes of patients who underwent coronary artery bypass grafting By Zhao Y, Zhang H, Wang X and Liu D. (2023) Front. Cardiovasc. Med. 10:1036285. doi: 10.3389/fcvm.2023.1036285

In the published article, there was an error in the order of the Funding statement. The original text “Thanks to the Beijing Municipal Science & Technology Commission for the financial support (no. Z201100005520038). Also to National High Level Hospital Clinical Research Funding (2022-PUMCH-B-026)” has been corrected. The new version appears below.

