# Supplementary material for: Impact of central venous pressure during the first 24 h and its time-course on the lactate levels and clinical outcomes of patients who underwent coronary artery bypass grafting
Source: Front Cardiovasc Med. 2023 May 23;10:1036285. doi: 10.3389/fcvm.2023.1036285 (PMC10269904; doi:10.3389/fcvm.2023.1036285)

Table S1: Baseline characteristics of the patients included in this study

| Covariates | Patients included in this study (n=5552) |
| --- | --- |
| N | 5552 |
| Age | 69 (62-76) |
| Male (%) | 4285/5552 (77.2) |
| Weight (kg) | 84.4 (73.4-97.0) |
| Ethnicity, n (%) |  |
| Asian | 131/5552 (2.4) |
| Black | 201/5552 (3.6) |
| White | 4005/5552 (72.1) |
| Latino | 165/5552 (3.0) |
| Other | 1050/5552 (18.9) |
| Comorbidities, n (%) |  |
| Myocardial infarction | 2196/5552 (39.6) |
| Congestive heart failure | 1405/5552 (25.3) |
| Diabetes | 2344/5552 (42.2) |
| Liver disease | 209/5552 (3.8) |
| Chronic renal disease | 1029/5552 (18.5) |
| Hypertension | 3370/5552 (60.7) |
| Atrial fibrillation | 2262/5552 (40.7) |
| Severity of illness |  |
| SOFA score | 5 (4-7) |
| SAPS II score | 36 (30-43) |
| Charlson comorbidity index | 5 (4-7) |
| Vital signs |  |
| MAP (mmHg) | 58 (53-62) |
| Heart rate (bpm) | 68 (61-74) |
| Temperature (°C) | 36.3 (35.6-36.4) |
| Respiratory rate (bpm) | 11 (9-13) |
| Laboratory tests |  |
| WBC (× 10^9^/L) | 9.1 (7.0-12.6) |
| Hemoglobin (× 10^12^/L) | 11.5 (9.6-13.3) |
| Platelet (× 10^9^/L) | 182 (142-228) |
| Bicarbonate (mmol/L) | 24 (23-26) |
| Bun (mg/dL) | 17 (14-22) |
| Creatinine (mg/dL) | 0.90 (0.80-1.20) |
| Primary outcome |  |
| 28-day mortality | 76/5552 (1.4) |
| Secondary outcomes |  |
| In-hospital mortality | 84/5552 (1.5) |
| 1-year mortality | 134/5552 (2.4) |
| Length of ICU stay (days) | 1.88 (1.23-3.16) |
| Length of hospitalization (days) | 7.28 (5.35-10.65) |
| AKI within 7 days, n (%) | 1630/5552 (29.4) |
| RRT, n (%) | 105/5552 (1.9) |
| Vasopressor, n (%) | 4596/5552 (82.8) |
| Lactate level (mmol/L) | 1.2 (1.0-1.6) |
| Duration of ventilation (hours) | 5.60 (3.60-13.39) |
| PO2/FiO2 (mmHg) | 200 (142-265) |

Figure S1: Standardized mean difference (SMD) of variables before and after propensity score matching and weighting


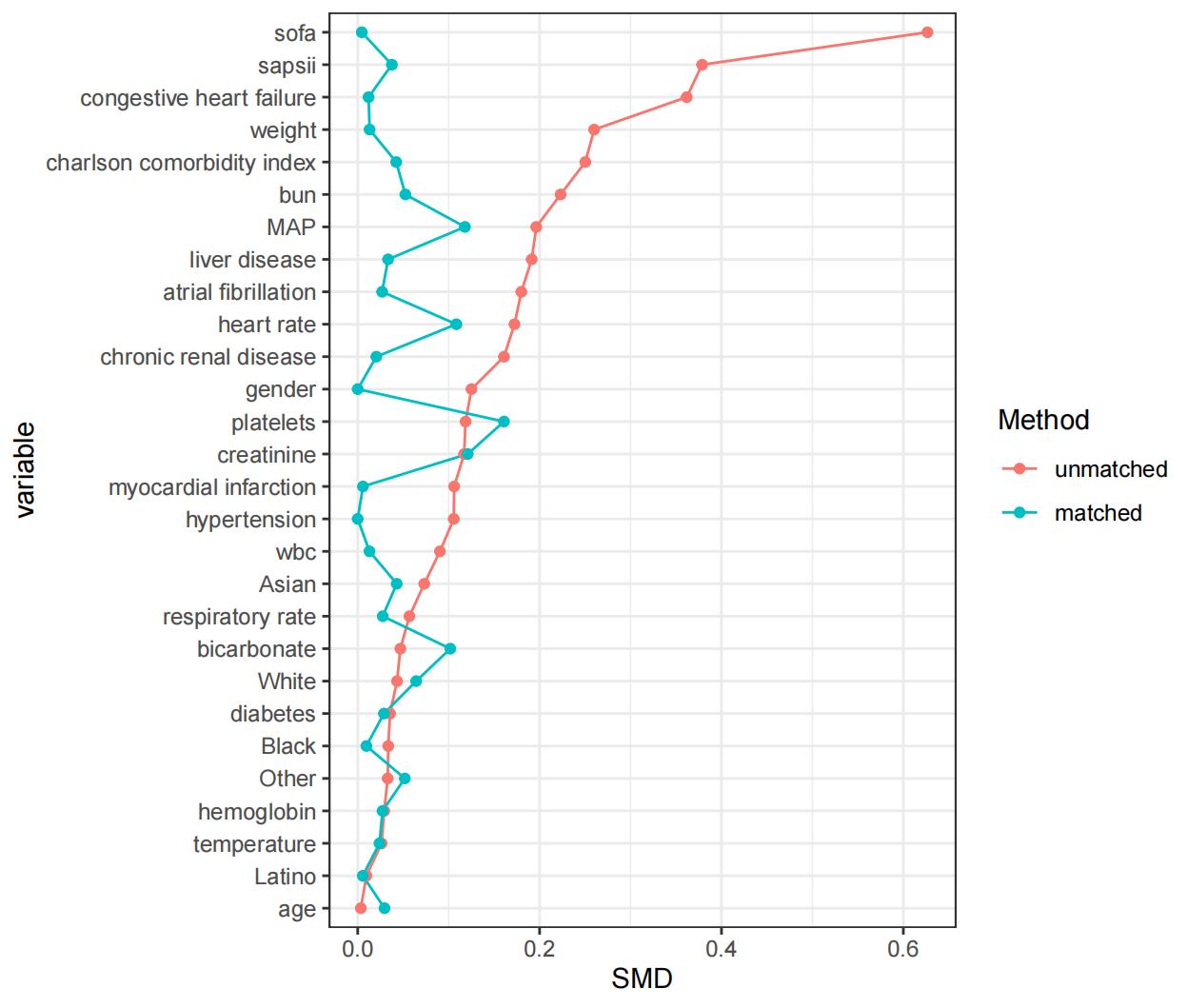

Supplement: Supplementary file 1 [file Datasheet1.docx]
